# The combination effect of homoharringtonine and ibrutinib on FLT3-ITD mutant acute myeloid leukemia

**DOI:** 10.18632/oncotarget.14463

**Published:** 2017-01-03

**Authors:** Xia Li, Xiufeng Yin, Huafeng Wang, Jiansong Huang, Mengxia Yu, Zhixin Ma, Chenying Li, Yile Zhou, Xiao Yan, ShuJuan Huang, Jie Jin

**Affiliations:** ^1^ Department of Hematology, The First Affiliated Hospital of Zhejiang University, Hangzhou, People's Republic of China; ^2^ Institute of Hematology, Zhejiang University School of Medicine, Hangzhou, People's Republic of China; ^3^ Key Lab of Hematopoietic Malignancy, Zhejiang University, Hangzhou, Zhejiang, People's Republic of China

**Keywords:** acute myeloid leukemia, FLT3-ITD, homoharringtonine, ibrutinib

## Abstract

Acute myeloid leukemia (AML) is a highly heterogeneous disease and internal tandem duplication mutation in FMS-like tyrosine-kinase-3 (FLT3-ITD) has a negative impact on outcome. Finding effective treatment regimens is desperately needed. In this study, we explored the inhibitory effect and mechanism of homoharringtonine (HHT) in combination with ibrutinib on FLT3-ITD mutant AML cells. Consequently, we observed a synergistic inhibitory effect when ibrutinib was combined with HHT to inhibit cell proliferation, induce apoptosis and arrest cell cycle at G0/G1 phase in MV4-11 and MOLM-13 leukemia cells. Our results indicate that the mechanisms of the combination effect are mainly via regulating the STAT5/Pim-2/C-Myc pathway, AKT pathway and Bcl-2 family, activating p21WAF1/CIP1 and inhibiting CCND/CDK complex protein. Interestingly, synergistic cytotoxicity of ibrutinib and HHT was dependent on both FLT3 and BTK. Here we provide a novel effective therapeutic approach for the treatment of AML patients with FLT3-ITD mutation.

## INTRODUCTION

Acute myeloid leukemia (AML) has achieved a high prevalence of complete remission (CR) with the gold standard for induction chemotherapy using daunorubicin and cytarabine [1, 2]. However, approximately 20%~30% AML patients harbor an internal tandem duplication mutation of the FMS-like tyrosine kinase receptor (FLT3-ITD mutation) that was considered to be unfavorable. The CR rate was lower and the overall and disease-free survival was shorter than non-FLT3-ITD AML patients [3–6]. Until now, effective treatment regimens for FLT3-ITD mutant AML were still lacking and represent an urgent need.

Homoharringtonine (HHT) is a natural alkaloid derived from trees of Cephalotaxus and has been studied and used in China for the treatment of hematological diseases for the past 30 years. The mechanisms are not very clear but it works by inhibiting protein synthesis [7, 8] by preventing the initial elongation step of protein synthesis via an interaction with the ribosomal A-site and reducing p-eIF4E levels [9]. This leads to a rapid loss of proteins with short half-lives such as c-Myc, Mcl-1 and CyclinD1. In *in vitro* and *in vivo* studies, HHT has been reported to act against AML [10–12], MDS [13–15] and CML [16–19] cells. In an open-label, randomized, controlled phase III study performed by our group, the homoharringtonine-based induction regimen HAA (homoharringtonine, cytarabine and aclarubicin) showed 73% of patients (150/206) with AML (non-acute promyelocytic leukemia (APL)) achieved CR, which was significantly higher than that in the DA (daunorubicin and cytarabine) group (61%, 125/205) [10]. Also in their study, 40 FLT3-ITD mutant patients were included and the HAA regimen showed good curative effect of treatment. Recently, as reported by Xu et al. [20], sorafenib in combination with low-dose homoharringtonine as a salvage therapy was successfully administrated and obtained CR in primary refractory FLT3-ITD mutant AML. In conclusion, regimen including HHT has been used as alternative valid front line chemotherapy for AML in China.

Recently, some groups have shown that the inhibitor of Bruton's tyrosine kinase (BTK) ibrutinib blocks AML cell proliferation, adhesion to bone marrow stromal cells as well as migration [21–23]. Recently, one study indicated that ibrutinib combined with ethacridine had a synergistic cytotoxicity to AML cells [24]. Interestingly, Pillinger et al. and Wu et al. found ibrutinib was particularly effective in inhibiting FLT3-ITD mutant AML cell survival [25, 26]. They have confirmed that ibrutinib blocks the FLT3 mutation signaling pathway and inhibits the expression of STAT5, ERK, AKT, and C-Myc to suppress FLT3-ITD mutant AML cell growth. All these observations helped to promote the application of ibrutinib to FLT3-ITD mutant AML.

In this study, our group combined HHT with ibrutinib in AML cell lines and primary AML cells to identify the synergistic effect. We mainly studied the mechanisms in FLT3-ITD mutant AML cells. Finally, we provided a novel combination strategy in FLT3-ITD mutant AML patients.

## RESULTS

### HHT and ibrutinib synergistically inhibited growth of AML cell lines and primary AML cells

**Figure 1:**
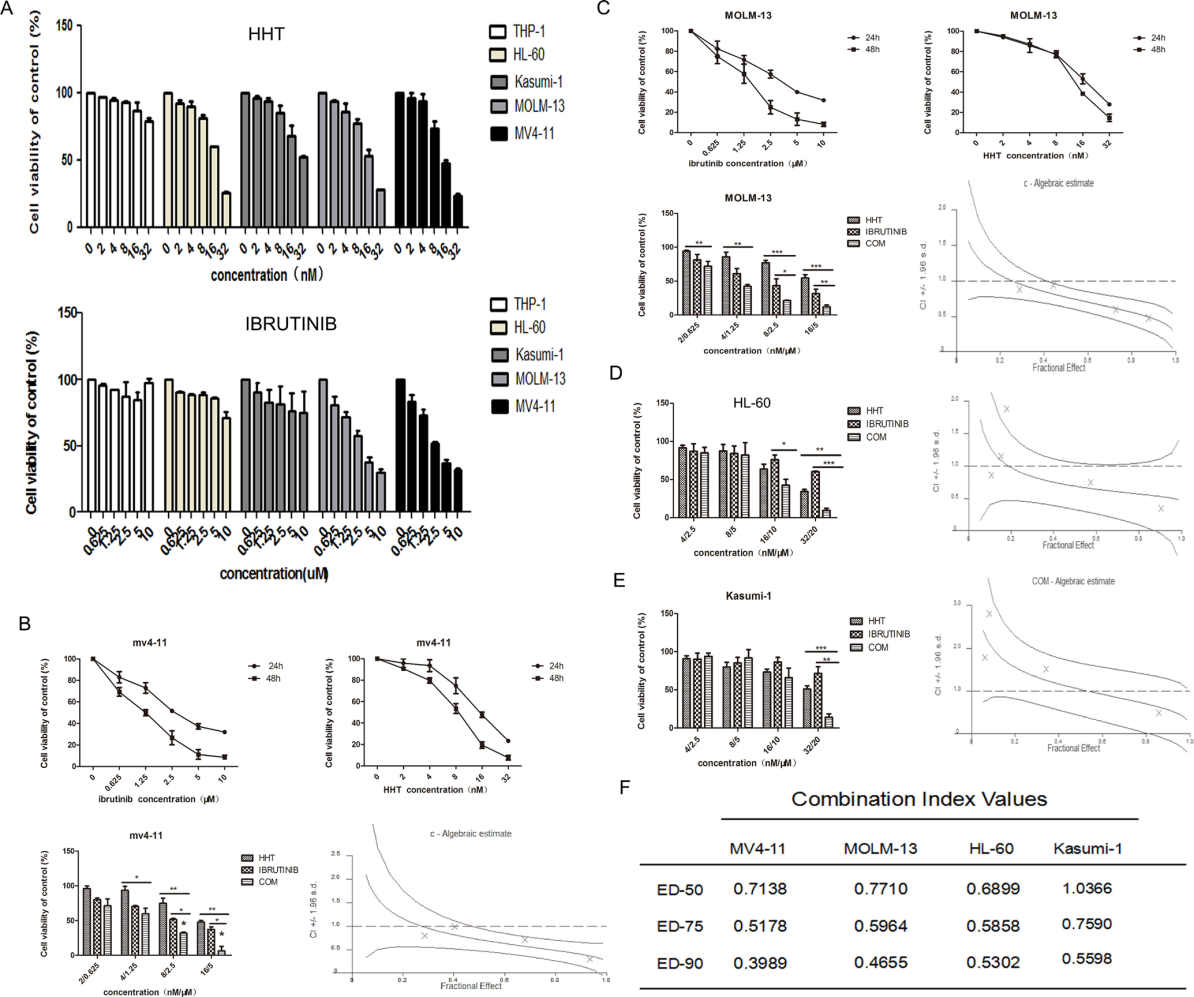
HHT and ibrutinib inhibit the growth of AML cell lines The cell viability induced by HHT and ibrutinib in AML cell lines at 24 h (**A**). The rate of cell viability induced by HHT, ibrutinib and HHT+ibrutinib in MV4-11cells (**B**), MOLM-13 cells (**C**), HL-60 cells (**D**) and Kasumi-1 cells (**E**) was measured by a MTT assay. The CI at the ED50, ED75 and ED90 were presented (**F**). The data are presented as mean inhibition rates ±SD from at least three independent experiments.

**Figure 2:**
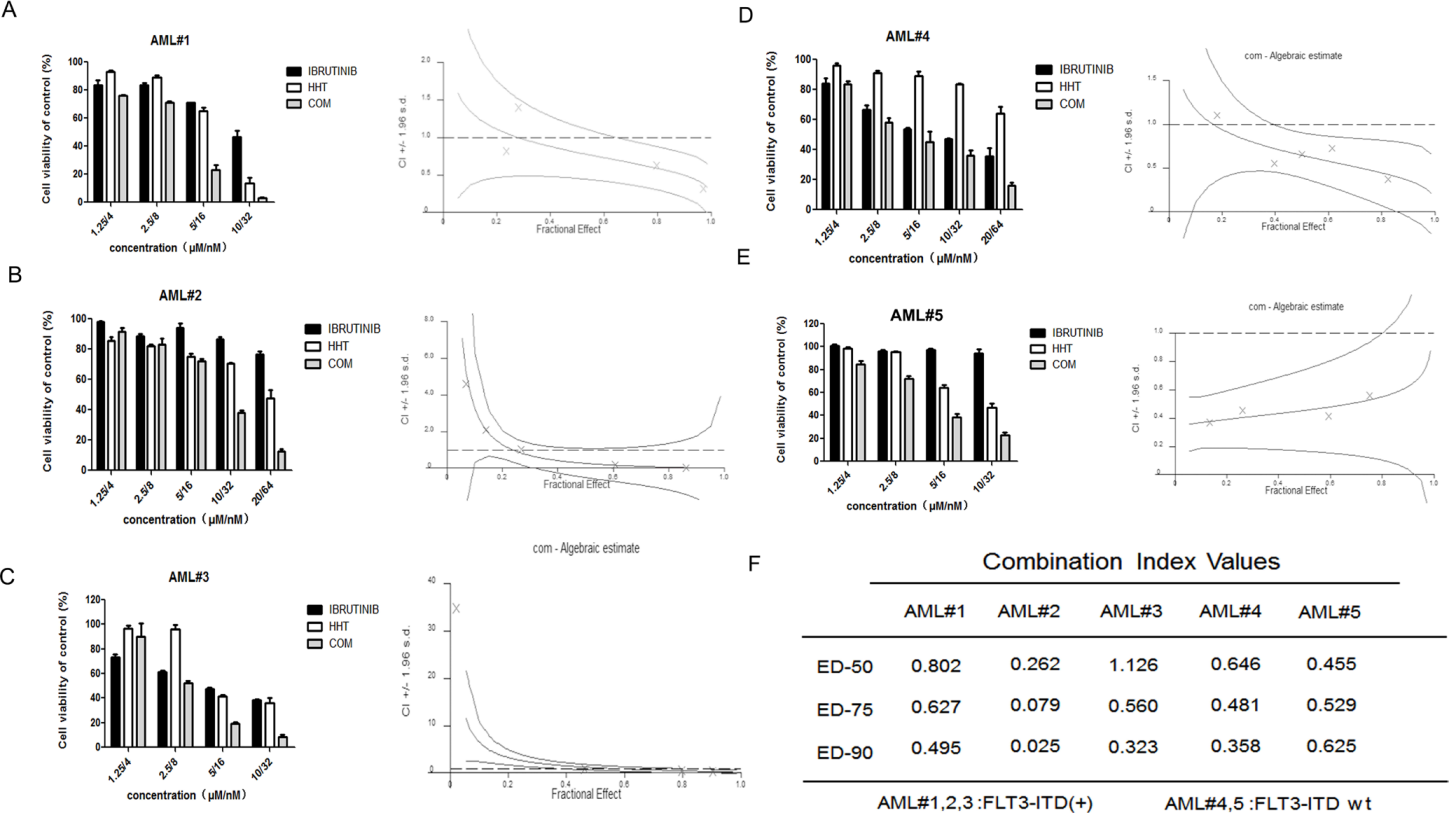
HHT and ibrutinib inhibit the growth of primary AML cells FLT3-ITD + primary AML cells (**A–D**) and FLT3-ITD wt primary AML cells (**E–G**) were treated with HHT, ibrutinib and HHT+ibrutinib for 24 h. The rate of cell viability was measured by an MTT assay. The CI at the ED50, ED75 and ED90 were presented (**H**).

The growth inhibition of AML cells by HHT or ibrutinib was examined. AML cells were plated into 96-well plates and treated with increasing concentrations of HHT (2, 4, 8, 16 and 32 nM) or ibrutinib (0.625, 1.25, 2.5, 5 and 10 μM) and equal volumes of DMSO for 24 h. Both HHT and ibrutinib showed higher sensitivity in MV4-11 and MOLM-13 cells (Figure 1A). As reported before, ibrutinib was more effective in inhibiting FLT3-ITD mutant AML cells [25, 27], similar to the findings in our study. The IC50s at 24 h for HHT and ibrutinib are presented in Supplementary Table 1. Following treatment with increasing doses of HHT or ibrutinib for 24 h and 48 h, MV4-11 and MOLM-13 cell viability was found to be significantly inhibited in a dose dependent manner. When HHT was combined with ibrutinib, they synergistically inhibited growth of AML cell lines (Figure 1) and similar results were achieved in primary AML cells (Figure 2). The characteristics of the patient samples are presented in Supplementary Table 2. The dose–effect curves were determined by Calcusyn analyses. CIs at the ED50, ED75 and ED90 are presented in Figures 1 and 2. CI < 1.0 means a synergistic effect. So we confirmed that HHT combined with ibrutinib had a strong synergistic effect in AML cell lines and primary AML cells *in vitro*.

### HHT and ibrutinib synergistically induced apoptosis of FLT3-ITD (+) AML cells

**Figure 3:**
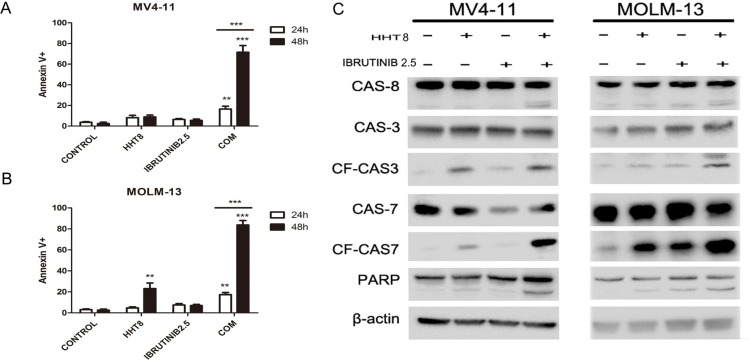
Apoptosis induced by HHT, ibrutinib and HHT + ibrutinib in FLT3-ITD mutant cells (**A**) MV4-11 and MOLM-13 cells (**B**) were treated with 8 nM HHT and/or 2.5 μM ibrutinib for 24 h and 48 h. Cells were co-stained with Annexin V and PI and apoptosis was measured by flow cytometry. Expression of PARP, caspase-3, -7 and -8 were analyzed by Western blotting analyses in MV4-11 and MOLM-13 cells treated with HHT and/or ibrutinib at the indicated concentrations for 24 h (**C**).

**Figure 4:**
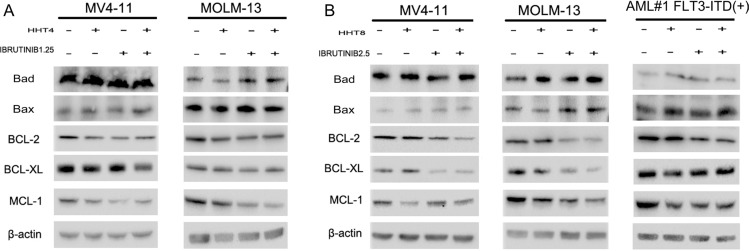
HHT combination ibrutinib inhibits BCL-2 family signaling (**A and C**) MV4-11 and MOLM-13 cells were treated with 4 nM HHT and/or 1.25 ibrutinib for 6 h. (**B and D**) MV4-11, MOLM-13 and primary AML cells were treated with 8 nM HHT and/or 2.5 μM ibrutinib for 6 h. Western blot analysis was conducted for p-Bad, Bad, Bax, Bcl-2, Bcl-xL and Mcl-1protein levels.

In this study, we investigated if these two drugs could synergistically induce apoptosis of FLT3-ITD mutant AML cells. Cells were exposed to 8 nM HHT and/or 2.5 μM ibrutinib for 24 h and 48 h. Compared with single agents, combination of HHT and ibrutinib resulted in a significant increase in apoptosis of MV4-11 and MOLM-13 cells (Figure 3A and 3B). Next, we analyzed the key signaling molecules in the apoptosis pathway by western blot analysis. As presented in Figure 3C, we observed that the combined treatment obviously increased the expression of cleaved PARP, caspase 8, caspase 7 and caspase 3 at 24 h. The expression of apoptotic proteins Bad and Bax had no obvious change. The expression of main anti-apoptotic proteins including Bcl-2, Bcl-XL and MCL-1 were more significantly decreased caused by HHT combined with ibrutinib after 6 h exposure (Figure 4A and 4B).

### HHT combined with ibrutinib enhanced cell cycle arrest

**Figure 5:**
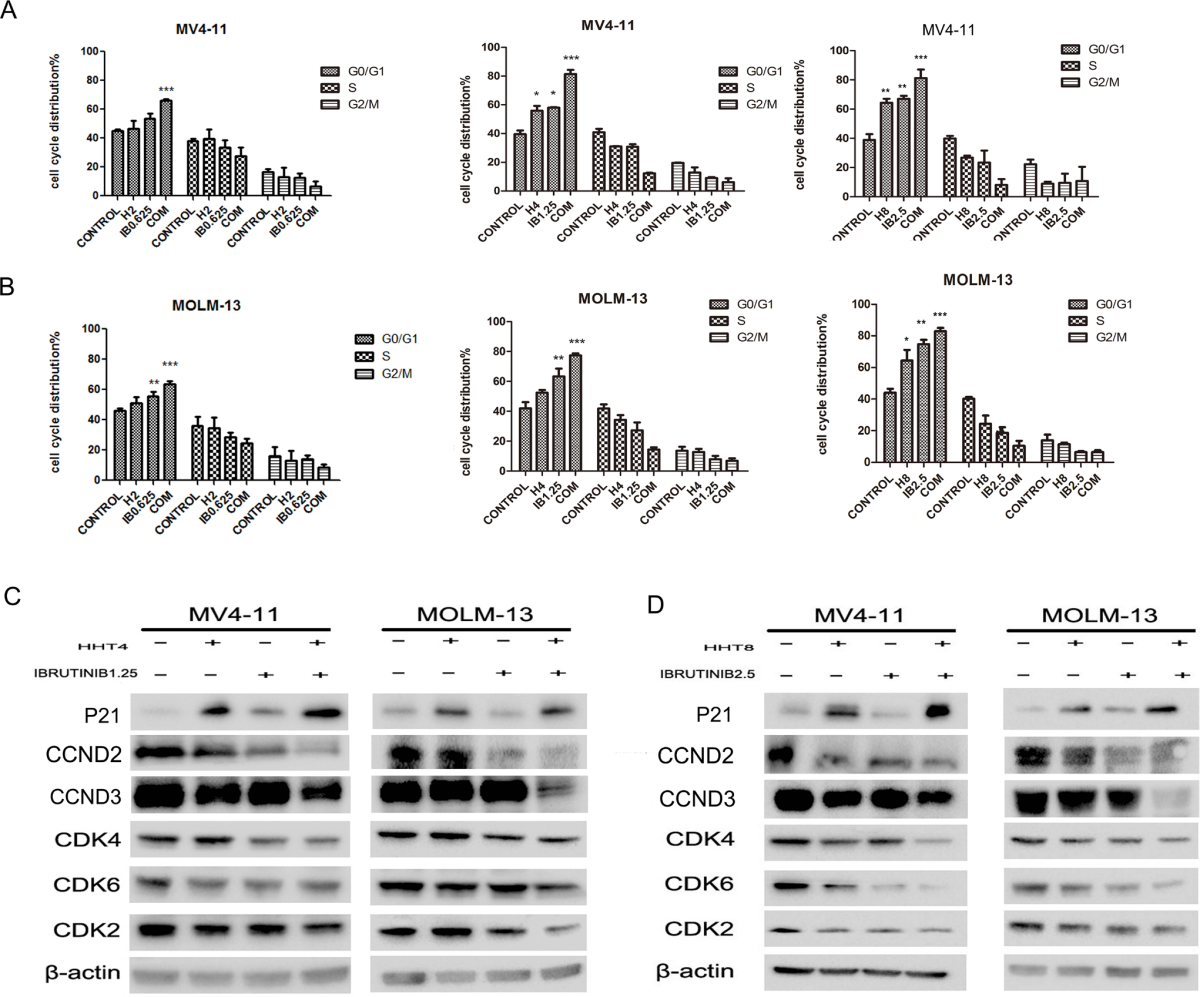
Effects of HHT, ibrutinib, HHT+ibrutinib on cell cycle distribution in AML cells (**A**) MV4-11 and MOLM-13 cells were treated with 4 nM HHT and/or 1.25 μM ibrutinib for 24 h. (**B**) MV4-11 and MOLM-13 cells were treated with 8 nM HHT or/and 2.5 μM ibrutinib for 24 h. The cells were stained with propidium iodide and subjected to flowcytometry analysis to determine cell cycle distribution. (**C and D**) Soluble proteins P21, CCDN2, CCDN3, CDK4, CDK6, CDK2 and β-actin were analyzed by Western blotting analyses at the indicated concentrations for 24 h.

Cell cycle arrest mainly contributed to the synergistic effects of HHT and iburutinib in FLT3-ITD mutant AML cells. Treated MV4-11 and MOLM-13 cells with 2 nM HHT or 0.625 μM ibrutinib did not cause cell cycle arrest, but the group 2+0.625 caused G0/G1 arrest (Figure 5A). When cells were treated with 4 nM HHT and/or 1.25 μM ibrutinib, we found both single agents and combination apparently caused G0/G1 arrest (*p* < 0.01) (Figure 5B). We then increased the concentration of the two agents to 8 nM and 2.5 μM. Cell cycle distribution at G0/G1 phase increased, but the group of 8+2.5 was almost the same as the 4+1.25 group (Figure 5C). This leads us to believe that when HHT is combined with ibrutinib at low concentrations the synergistic effects were via cell cycle arrest and at high concentrations the combination also caused apoptosis as showed in Figure 3. To further prove the induction of cell cycle arrest, we analyzed the expression level of G1 proteins cyclin D2/D3 (CCND2/3) and G1 phase enhancers cyclin dependent kinase 4, 6, 2 (CDK-4, CDK-6, CDK-2) and found obvious deceases in both low concentration and high concentration groups (Figure 5D). We found that HHT mainly down-regulated CCND3 and ibrutinib mainly down-regulated CCND2 and both CCND2 and CCND3 were obviously down-regulated in combination group. We also analyzed the cyclin dependent kinase inhibitor (CDKI) P21WAF1/CIP1. Interestingly, HHT caused a significant increase of P21WAF1/CIP1 which was not reported before. As expected, the combination of HHT and ibrutinib induced a more pronounced up-regulation of P21WAF1/CIP1 expression.

### The synergistic effect increased p53 through DNA damage response

**Figure 6:**
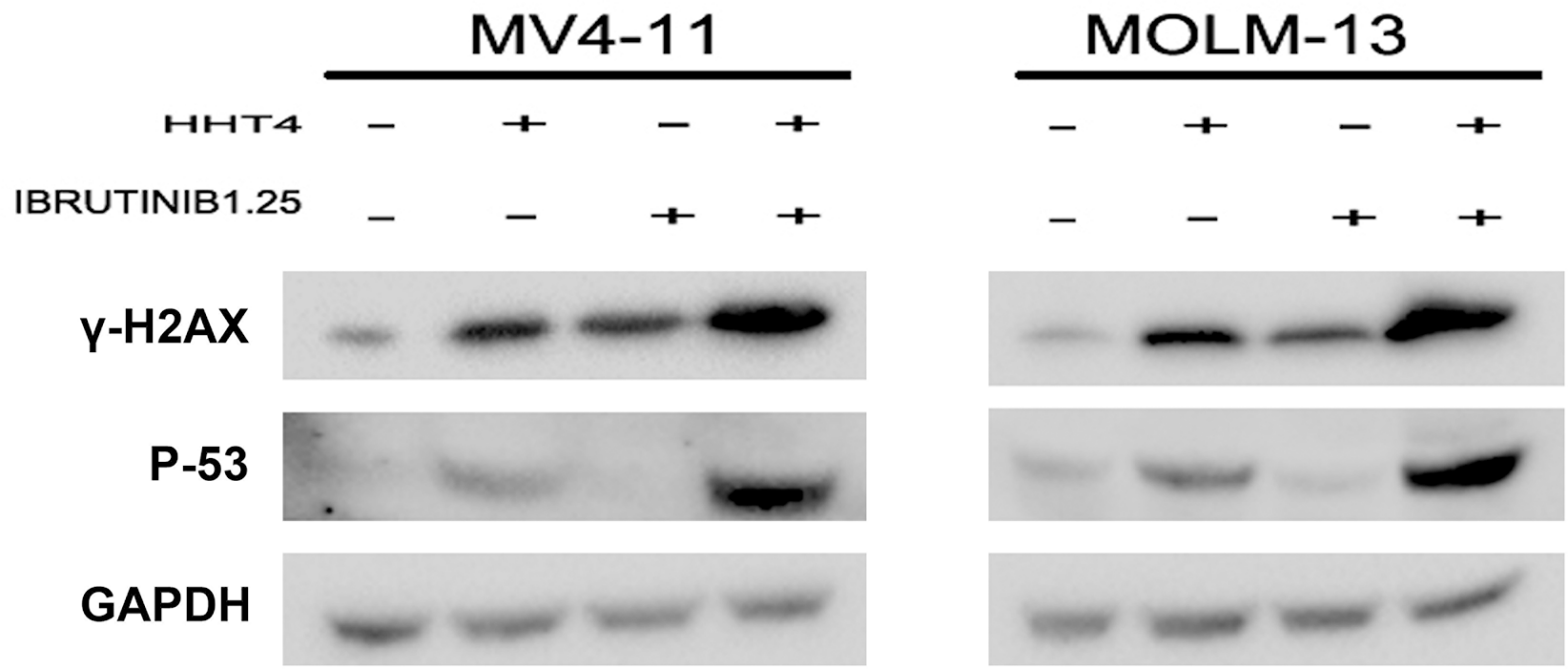
The synergistic effects increased γH2AX and p53 The protein levels of γH2AX and p53 were examined by western blot.

P53 is the upstream of P21WAF1/CIP1. In our study, we found that HHT obviously increased P53 and the combination of HHT and ibrutinib resulted in a more pronounced increase of P53 on the protein level. In order to investigate whether the up-regulation of P53 was dependent on DNA damage, we examined the level of γH2AX. As shown in Figure 6, we found the combination group increased γH2AX more significantly than single agents. These data indicated that the up-regulation of P53/P21 was dependent on DNA-damage response.

### Modulation of oncogenic signaling pathways by HHT and ibrutinib

**Figure 7:**
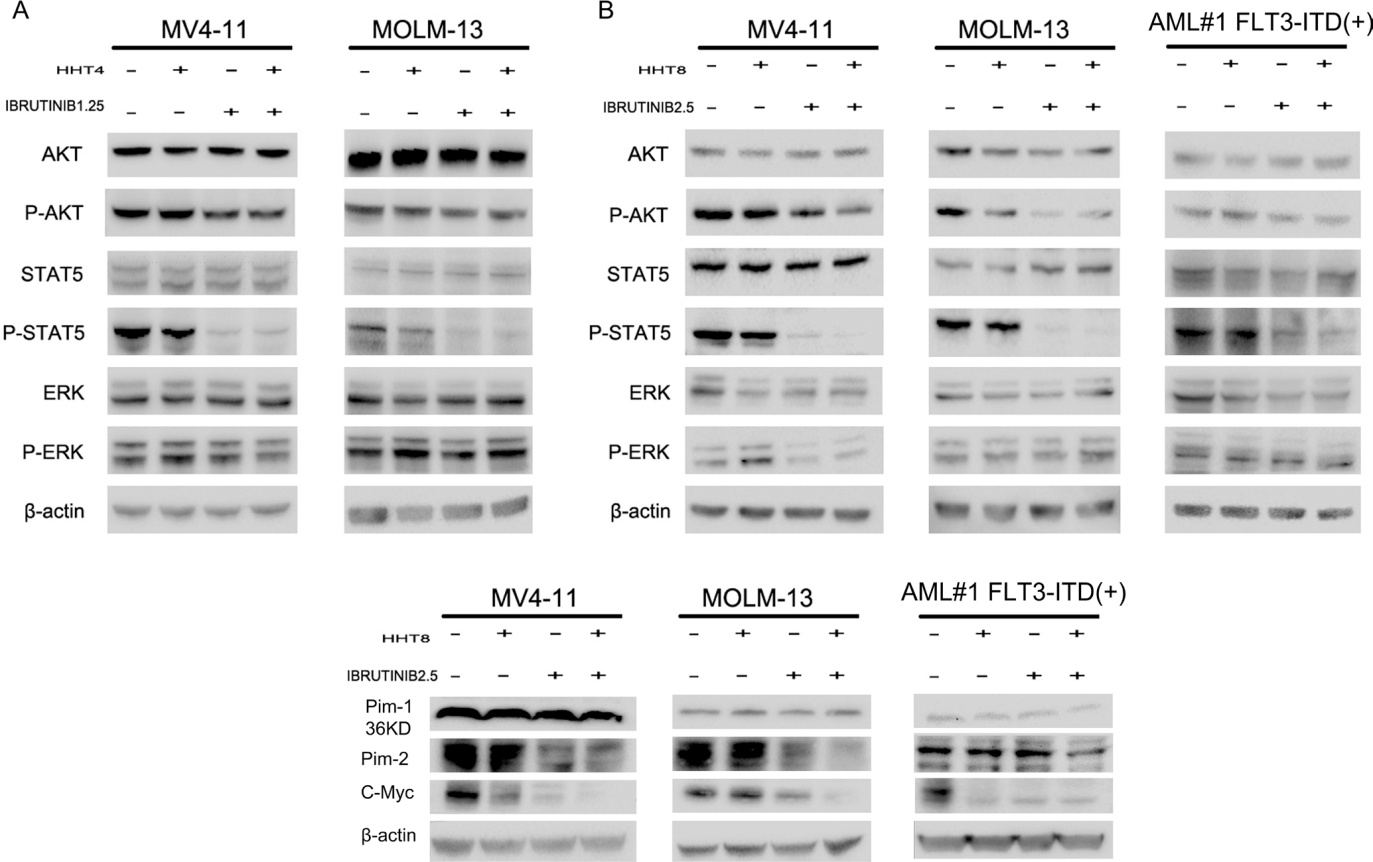
HHT combination ibrutinib inhibits STAT5, AKT signaling (**A and C**) MV4-11 and MOLM-13 cells were treated with 4 nM HHT and/or 1.25 ibrutinib for 6 h. (**B and D**) MV4-11, MOLM-13 and primary AML cells were treated with 8 nM HHT or/and 2.5 μM ibrutinib for 6 h. Western blot analysis was conducted for p-AKT-S473, total AKT, p-STAT5, STAT5, p-ERK, ERK, Pim-1, Pim-2 and C-Myc protein levels.

FLT3-ITD mutation leads to aberrant activation of downstream kinases including MAPK, AKT and STAT5 [25, 26]. In our study, we found the expression of phosphorylated STAT5 (p-STAT5) was notably reduced when treated with ibrutinib and the combination of HHT and ibrutinib for 6 h in MV4-11 and MOLM-13 cell lines (Supplementary Figure 1, Figure 7A and 7B). However, no difference was observed in phosphorylated ERK (p-ERK). When the concentration was increased to 8 nM HHT and/or 2.5 μM ibrutinib, phosphorylated AKT (p-AKT) was decreased as well. Similar results in a primary AML patient were shown in Figure 7B. Next, we observed Pim kinases were regulated by STAT5, down-stream of FLT3-ITD, and found Pim-2 was obviously decreased while Pim-1 had no change. Previously, researchers have reported both HHT and ibrutinib can inhibit transcription factor C-Myc [11, 27]. This led us to measure the expression of C-Myc and found an obvious inhibition when treated with HHT and ibrutinib (Figure 7C).

**Figure 8:**
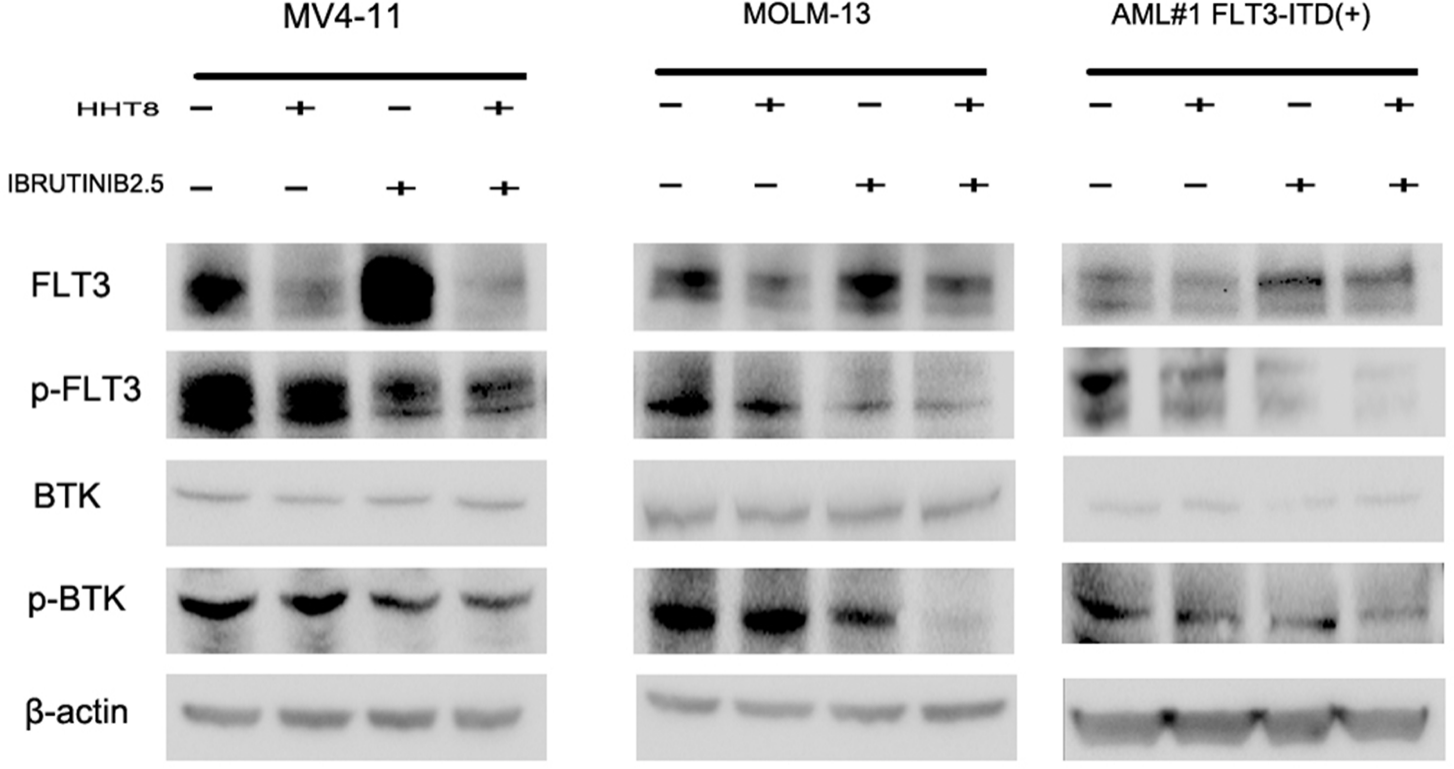
The level of main target proteins were analyzed when cells were exposed to drugs for 24 h MV4-11, MOLM-13 and primary AML cells were treated with 8 nM HHT and/or 2.5 μM ibrutinib for 24 h. Western blot analysis was conducted for FLT3, p-FLT3, BTK, and p-BTK223 protein levels.

The inhibitory effect of HHT combined with ibrutinib was through the down-regulation of both FLT3 and BTK pathways. To identify the effect of ibrutinib and/or HHT on FLT3 and BTK activity, we used western blot to analyze the protein levels of FLT3, p-FLT3, BTK and p-BTK (Y223) in MV4-11, MOLM-13 and FLT3-ITD (+) patients’ cells. As a result, we found HHT decreased both total FLT3 and p-FLT3, while ibrutinib decreased p-FLT3 significantly and increased total FLT3 simultaneously. For the combination of HHT and ibrutinib, both total FLT3 and p-FLT3 were obviously decreased. Meanwhile, the combination group caused a more significant decrease in the expression of p-BTK after 6 h exposure (Figure 8).

### Ibrutinib's synergy with HHT is dependent of FLT3

**Figure 9:**
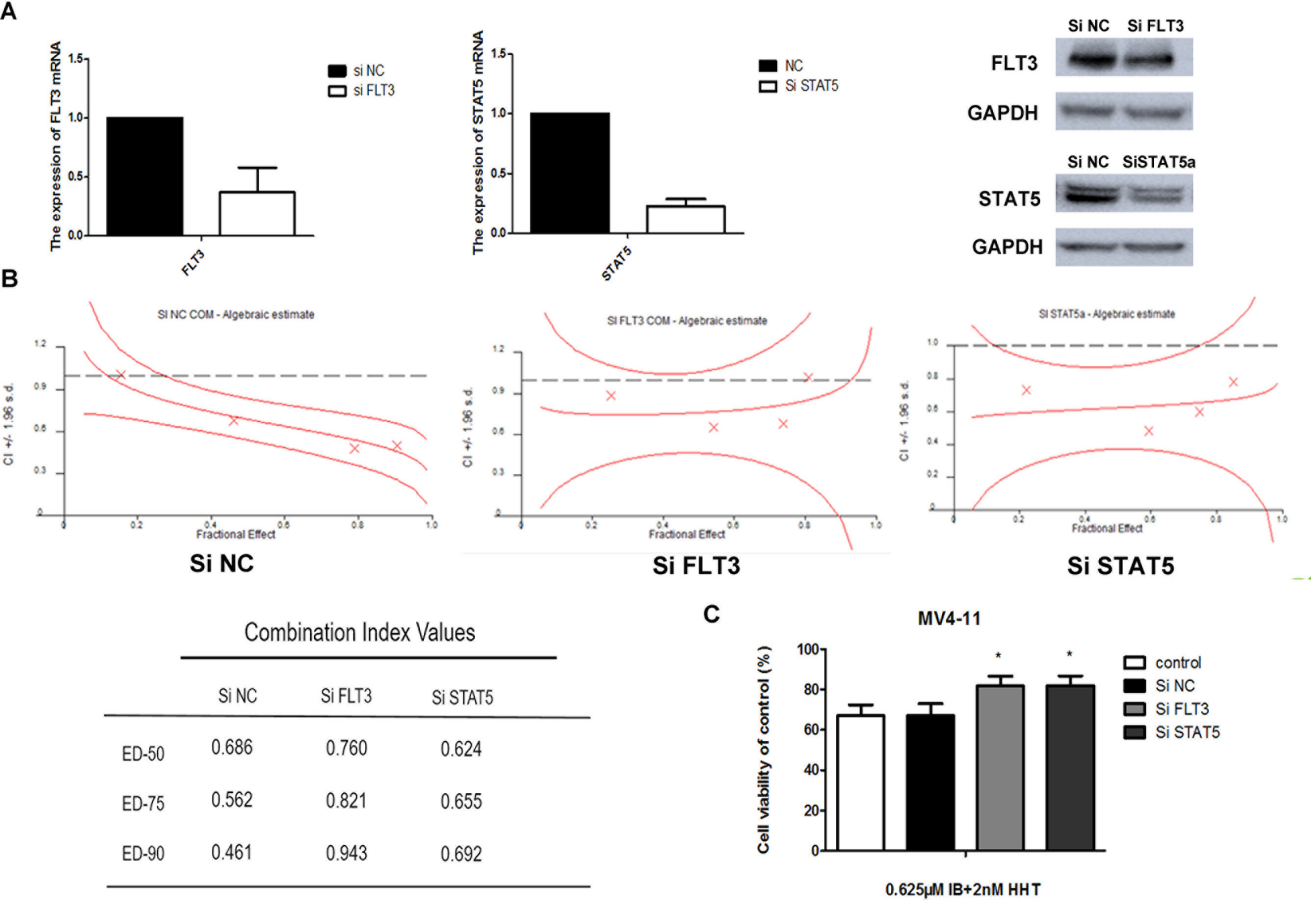
Ibrutinib's synergy with HHT is dependent of FLT3 MV4-11 cells were transduced with 3 different SiRNAs targeting FLT3, STAT5 or a non-targeting siRNA control. The reduction of FLT3 or STAT5 expression was confirmed by qPCR and western blotting. (**A**) On day 3 to 5 post-transduction, cells were treated with HHT at concentrations previously shown to synergize with ibrutinib for 24 h. The CIs at the ED50, ED75 and ED90 were presented. (**B**) Cells were treated with 0.625 μM ibrutinib and 2 nM HHT for 48 h. Cell growth and viability was determined by a MTT assay. (**C**) The data are presented as mean inhibition rates ± SD from at least three independent experiments.

To test the synergistic effects between ibrutinib and HHT whether due to interfere to FLT3 or STAT5, we knocked down the expression of the *FLT3* or *STAT5* gene in MV4-11 cells using siRNA technology. The reduction of FLT3 or STAT5 expression was confirmed by qPCR and western blotting (Figure 9A). Knockdown cells then were treated with increasing concentrations of ibrutinib and HHT for 24 h. CIs at the ED50, ED75 and ED90 are presented at Figure 9B. We found that there was almost no difference between STAT5-knockdown cells and scrambled siRNA control cells. The CIs of FLT3 knockdown cells were increased compared to the scrambled siRNA control cells. When cells were treated with 0.625 μM ibrutinib and 2 nM HHT for 48 h, compared to control siRNA treatment, the cell viability was increased in FLT3 or STAT5-knockdown cells (*P* < 0.05) (Figure 9C). These observations suggest that synergy of ibrutinib with HHT is dependent of FLT3 but not STAT5.

## DISCUSSION

The treatment for FLT3-ITD mutant AML patients remains a seriously unmet medical need. Besides standard chemotherapy, there are several inhibitors such as sorafenib [31, 32], midostaurin [33], lestaurtinib [34] and quizartinib [35, 36] that have been used clinically to date. However, drug resistance frequently develops within the first 12 months of treatment. Our previous studies found that chemotherapy regimen including HHT had a powerful effect in FLT3-ITD mutant patients [10]. According to the results of particularly effective effect in inhibiting FLT3-ITD mutant AML cells survival of ibrutinib, we assumed a synergistic effect between HHT and ibrutinib. Here, we mainly evaluated the effects of single agents and the combination of two agents in FLT3-ITD mutant cell lines and primary cells from FLT3-ITD mutant patients. The probable mechanisms of synergistic effect were also explored.

In this study, we showed that HHT or ibrutinib alone induced an inhibition of cell growth in MV4-11 and MOLM-13 cells in a dose-dependent manner. Both HHT and ibrutinib showed higher sensitivity in FLT3-ITD+ cells than FLT3-ITD wt cells. When combining HHT with ibrutinib at the molar rate 1:312.5 (HHT nM: ibrutinib nM), a synergistic effect was significantly seen in ED50, 75, 90 identified by calculation of CI. Besides AML cell lines, primary AML blasts similarly showed a synergistic effect but we found no significant difference between FLT3-ITD+ and FLT3-ITD wt cells. In order to find effective treatment regimens for FLT3-ITD+ patients, FLT3-ITD+ AML cells were used in most experiments for exploring the mechanisms of the synergistic effect.

From our study, we found that the combination therapy enhanced apoptosis and cell cycle arrest, which might be the key factor of the synergistic effect mechanisms. To further elucidate the mechanisms involved in the synergistic effect, western blotting was used to identify the pathway modulated by HHT and ibrutinib.

As previously reported, HHT decreased protein expression levels of PI3K110 and P-AKT in THP-1 and Kasumi cells [11], and possibly acted as a broad-spectrum PTK inhibitor and inhibited JAK2-STAT5 signaling pathway in AML cells [37]. According to our results, HHT was found to slightly down regulate the expression of AKT and STAT5 signaling pathways at high concentration but not ERK in FLT3-ITD mutant AML cells. In addition, we firstly found that HHT up-regulated the levels of P53 and P21WAF1/CIP1. Most importantly, HHT obviously decreased not only p-FLT3 but also total FLT3.

The pharmacological mechanisms by which ibrutinib inhibits FLT3-ITD mutant AML cells are more legible. A recent study by Pillinger et al. demonstrated that BTK was activated in the downstream of the FLT3-ITD in AML and that ibrutinib induced survival and proliferation pathways including AKT, STAT5 and MAPK via targeting FLT3-ITD [25]. Our results were almost consistent. However, no obvious effect on MAPK pathway at 6 h point was observed in our study. For the mechanisms of synergistic effect, we found that HHT combined with ibrutinib induced simultaneous up-regulation of PARP, Caspases, γH2AX, P53, P21 WAF1/CIP1 and down-regulation of CCND2, CCND3, CDK4, 6, 2, P-AKT, P-STAT5/Pim-2/C-Myc and Bcl-2 family at the protein level in FLT3-ITD mutant AML cells. Supplementary Figure 2 summarized the pathway for combination mechanisms. Above all, for the combination of HHT and ibrutinib, it obviously decreased both total FLT3 and p-FLT3. Meanwhile, the combination group caused a more significant decrease in the expression of p-BTK. The combination of HHT and ibrutinib simultaneously inhibited first the activity of FLT3 and BTK and then STAT5 and C-Myc. These processes caused DNA damage, induced P53 overexpression, activated P21, and then triggered cell cycle arrest and apoptosis. This phenomenon was highlighted in FLT3-ITD+ cells. However, the combination group still can't inhibit P-ERK. Therefore, further studies are of great necessity to combine another antineoplastic agent to down regulate ERK pathway and explore a more effective chemotherapy regimen.

Therefore, we conclude that co-treatment with HHT and ibrutinib is a potentially new therapeutic regimen for FLT3-ITD mutant AML and further clinical studies are required.

## MATERIALS AND METHODS

### Materials

Anti-phosphorylated and total FLT3, BTK (223), AKT, STAT5 and ERK, CCDN2, CCND3, CDK2, CDK4, CDK6, P53, γH2AX, P21WAF1/CIP1, CASPASE-3, CASPASE-7, CASPASE-8, PARP, Bad, Bax, Bcl-2, Bcl-XL, MCL-1, Pim-2, γ-H2AX, and β-actin antibodies were purchased from Cell Signalling Technology (Beverly, MA, USA). Pim-1 was obtained from Abcam (Cambridge, MA). Ibrutinib was obtained from Selleck Chemicals (Houston, USA). HHT was purchased from Sigma-Aldrich (St. Louis, MO, USA).

### Methods

### Cell lines and primary cells

The FLT3-ITD+ AML cell lines MV4-11 and MOLM-13 were kindly endowed by Professor Ravi Bhatia (City of Hope National Medical Center, Duarte, CA). These two cell lines were cultured in IMDM medium (Gibco, Billings, MT, USA) supplemented with 10% fetal bovine serum (Gibco) at 37°C in a humidified incubator containing 5% CO2. Bone-marrow samples were obtained from AML patients after obtaining written informed consent. Peripheral blood mononuclear cells (PBMCs) were isolated by Ficoll-Hypaque (Sigma–Aldrich) density gradient centrifugation. The mutations in FLT3 internal tandem duplication (FLT-3ITD) were tested by the First Affiliated Hospital (Zhejiang University College of Medicine). The study was approved by the Ethics Committee of the First Affiliated Hospital, College of Medicine, Zhejiang University (Hangzhou, China).

### Growth inhibition assay

Cells were seeded in 96-well plates at 1 × 10^5^ (cell-line cells) or 5 × 10^5^ (primary AML cells). After treated with different drugs for 24 h, 20 ul MTT solution (5 mg/mL) was added to each well and the cells were incubated for an additional 4 h at 37°C. The cell medium was then removed and 200 ul DMSO was added to the 96-well plates to dissolve the MTT crystals. The plates were read at an absorbance of 570 nm. Cell-lines experiments were repeated three times.

### Apoptosis assay

Cells were treated with drugs for 24 h and 48 h and then harvested. After washed twice with phosphate buffered saline (PBS), cells were resuspended in binding buffer. Cells were then co-stained with 5 ul AnnexinV-Fluorescein Isothiocyanate (FITC) and 5 ul Propidium Iodide (PI) using an apoptosis detection kit (BD Pharmingen, San Diego, CA, USA). The apoptic cells were analyzed by FACScan flow cytometer (Becton Dickinson, San Diego, CA, USA).

### Cell cycle analysis

Cells were treated with drugs for 24 h. At the end of the treatment, the cells were washed with PBS and fixed with 75% ethanol at 4°C. The next day, the cells were harvested and washed with PBS twice then resuspended in buffer with 50 μg/ml PI (propidium iodide) and 100 μg/ml RNase A for 30 min. The DNA content was analysed by FACScan flow cytometer (Becton Dickinson, San Diego, CA, USA).

### Western blot analysis

Cells from various conditions were harvested and washed twice in PBS and lysed a RIPA buffer (Cell Signaling Technology, Beverly, MA, USA) on ice for 30 min. Cells were then centrifuged at 12000g for 15 min at 4°C and the supernatant was collected. The protein concentration was determined using BCA reagent. Protein samples were separated via 10% SDS-PAGE gel (Life Technology, USA) and transferred to a PVDF membrane (Millipore, Billerica, MA, USA). Next, the membranes were blocked in Tris-buffered solution (TBS) containing 5% non-fat milk for 1 h and incubated with primary antibodies overnight at 4°C. After washed with TBS-T buffer three times, membranes were incubated with secondary antibodies (CST, Beverly, MA, USA) for 1.5 h. The target protein bands were visualized using an ECL kit (Thermo scientific, USA) and analysis by the image lab software (bio-rad, california, USA)

### Real-time reverse transcription-polymerase chain reaction (RT-PCR)

Total RNA was extracted using TRIzol (Invitrogen, Carlsbad, CA, USA). About 500 ng of total RNA was used for reverse transcription reaction. Quantitative PCR was performed in triplicate using SYBR-Green PCR Master Mix kit (Takara, Japan) on an IQ5 real time PCR instrument (Bio-Rad, USA). The primers sequences were as follows: FLT3 5′-CTGAATTGCCAGCCACATTTTG-3′ (forward) and 5′-GGAACGCTCTCAGATATGCAG-3′ (reverse); STAT5 5′-GCAGAGTCCGTGACAGAGG-3′ (forward) and 5′-CCACAGGTAGGGACAGAGTCT-3′ (reverse); GAPDH 5′-TCAACGACCACTTTGTCAAGCTCA-3′ (forward) and 5′-GCTGGTGGTCCAGGGGTCTTACT-3′ (reverse).

### RNA interference

FLT3 and STAT5 siRNA were purchased from Novars (Shenzhen, china). Transfection was performed using LipofectamineTM RNAiMAX (Invitrogen) according to the manufacturer's instructions. MV4-11 cells were seeded at 5 × 10^5^/well in 6-well plates. A total of 200 nM of each siRNA and 10 ul Lipofectamine were mixed in 250 ul Opti-MEM (Invitrogen). After incubation at room temperature for 10 min, the siRNA–lipid complex was added to cells, incubated at 37°C for 72 h and then cells were collected for RT-PCR, Western blotting and MTT assay.

### Statistical analysis

The combination index (CI) was calculated using CalcuSyn software (Biosoft, Cambridge, UK). Student's *t*-test was used to assess statistical significance. Results with *P* < 0.05 were considered to indicate a statistically significant difference (*). Results represented the mean ± SD of 3 independent experiments.

## SUPPLEMENTARY MATERIALS TABLES AND FIGURES


