# Correction: The combination effect of homoharringtonine and ibrutinib on FLT3-ITD mutant acute myeloid leukemia

**DOI:** 10.18632/oncotarget.27310

**Published:** 2019-11-12

**Authors:** Xia Li, Xiufeng Yin, Huafeng Wang, Jiansong Huang, Mengxia Yu, Zhixin Ma, Chenying Li, Yile Zhou, Xiao Yan, ShuJuan Huang, Jie Jin

**Affiliations:** ^1^ Department of Hematology, The First Affiliated Hospital of Zhejiang University, Hangzhou, People’s Republic of China; ^2^ Institute of Hematology, Zhejiang University School of Medicine, Hangzhou, People’s Republic of China; ^3^ Key Lab of Hematopoietic Malignancy, Zhejiang University, Hangzhou, Zhejiang, People’s Republic of China

**This article has been corrected:** Due to errors in image processing, the β-actin bands for MOLM-13 cell line in Figure 5 (C and D) were mistakenly presented. The proper Figure 5 (C and D) is shown below. In addition, the figure legends of Figure 2, 4, 7, and 8 are incorrect. The correction figure legends of Figure 2, 4, 7, and 8 are listed below. The authors declare that these corrections do not change the results or conclusions of this paper.

Original article: Oncotarget. 2017; 8:12764–12774. 12764-12774. https://doi.org/10.18632/oncotarget.14463

**Figure 5:**
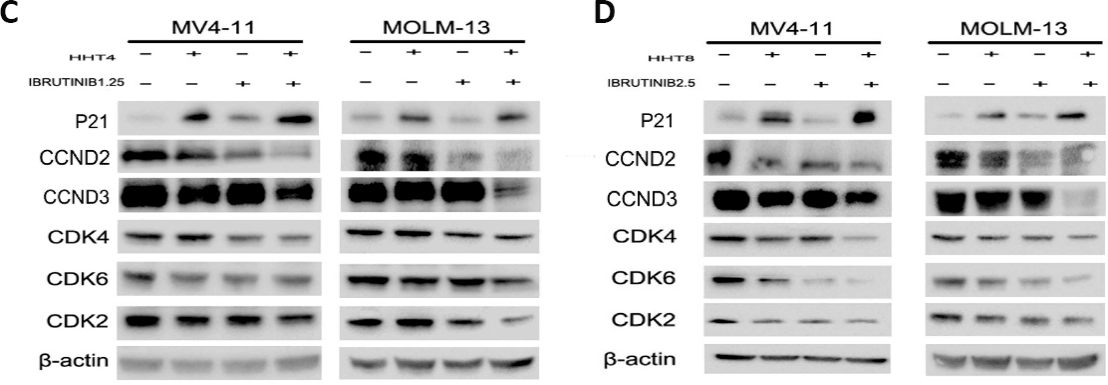
Effects of HHT, ibrutinib, HHT+ibrutinib on cell cycle distribution in AML cells. (**A**) MV4-11 and MOLM-13 cells were treated with 4 nM HHT and/or 1.25 μM ibrutinib for 24 h. (**B**) MV4-11 and MOLM-13 cells were treated with 8 nM HHT or/and 2.5 μM ibrutinib for 24 h. The cells were stained with propidium iodide and subjected to flowcytometry analysis to determine cell cycle distribution. (**C** and **D**) Soluble proteins P21, CCDN2, CCDN3, CDK4, CDK6, CDK2 and β-actin were analyzed by Western blotting analyses at the indicated concentrations for 24 h.

**Corrections of figure legends**

**Figure 2: HHT and ibrutinib inhibit the growth of primary AML cells.** FLT3-ITD + primary AML cells (**A**–**C**) and FLT3-ITD wt primary AML cells (**D** and **E**) were treated with HHT, ibrutinib and HHT+ibrutinib for 24 h. The rate of cell viability was measured by an MTT assay. The CI at the ED50, ED75 and ED90 were presented (**F**).

**Figure 4: HHT combined with ibrutinib inhibits BCL-2 family signaling.** (**A**) MV4-11 and MOLM-13 cells were treated with 4 nM HHT and/or 1.25 ibrutinib for 6 h. (**B**) MV4-11, MOLM-13 and primary AML cells were treated with 8 nM HHT and/or 2.5 μM ibrutinib for 6 h. Western blot analysis was conducted for p-Bad, Bad, Bax, Bcl-2, Bcl-xL and Mcl-1protein levels.

**Figure 7: HHT combined with ibrutinib inhibits STAT5, AKT signaling.** (**A**) MV4-11 and MOLM-13 cells were treated with 4 nM HHT and/or 1.25 ibrutinib for 6 h. (**B**) MV4-11, MOLM-13 and primary AML cells were treated with 8 nM HHT or/and 2.5 μM ibrutinib for 6 h. Western blot analysis was conducted for p-AKT-S473, total AKT, p-STAT5, STAT5, p-ERK, ERK, Pim-1, Pim-2 and C-Myc protein levels.

**Figure 8: The level of main target proteins were analyzed when cells were exposed to drugs for 6h.** MV4-11, MOLM-13 and primary AML cells were treated with 8 nM HHT and/or 2.5 μM ibrutinib for 6h. Western blot analysis was conducted for FLT3, p-FLT3, BTK, and p-BTK223 protein levels.

