# Supplementary material for: The combination effect of homoharringtonine and ibrutinib on FLT3-ITD mutant acute myeloid leukemia
Source: Oncotarget. 2017 Jan 3;8(8):12764–74. doi: 10.18632/oncotarget.14463 (PMC5355052; doi:10.18632/oncotarget.14463)
Supplement: Supplementary file 1 [file oncotarget-08-12764-s001.pdf]

The combination effect of homoharringtonine and ibrutinib on FLT3-ITD mutant acute myeloid leukemia

Supplementary Materials

Supplementary Table 1: The ic50 of HHT and ibrutinib in AML cell lines at 24 h

	THP-1	HL-60	Kasumi-1	MV4-11	MOLM-13
HHT nM	139.8	18.88	34.02	15.83	17.02
Ibrutinib µM	> 100	41.94	17.67	3.40	3.62

Supplementary Table 2: The characteristics of the patient samples

	Diagnose	Gender	Age (year)	FAB type	Cytogenetics	Molecular
AML#1	*de novo*	male	44	M5	46,XY	FLT3-ITD
AML#2	Relapse/refractory	male	68	M2a	complex karyotype	FLT3-ITD,CEBPA-N
AML#3	*de novo*	female	27	M1	46, XX	FLT3-ITD,NPM1,CEBPA
AML#4	*de novo*	male	47	M2	46, XY	–
AML#5	Relapse/refractory	female	45	M5b	46, XX	–

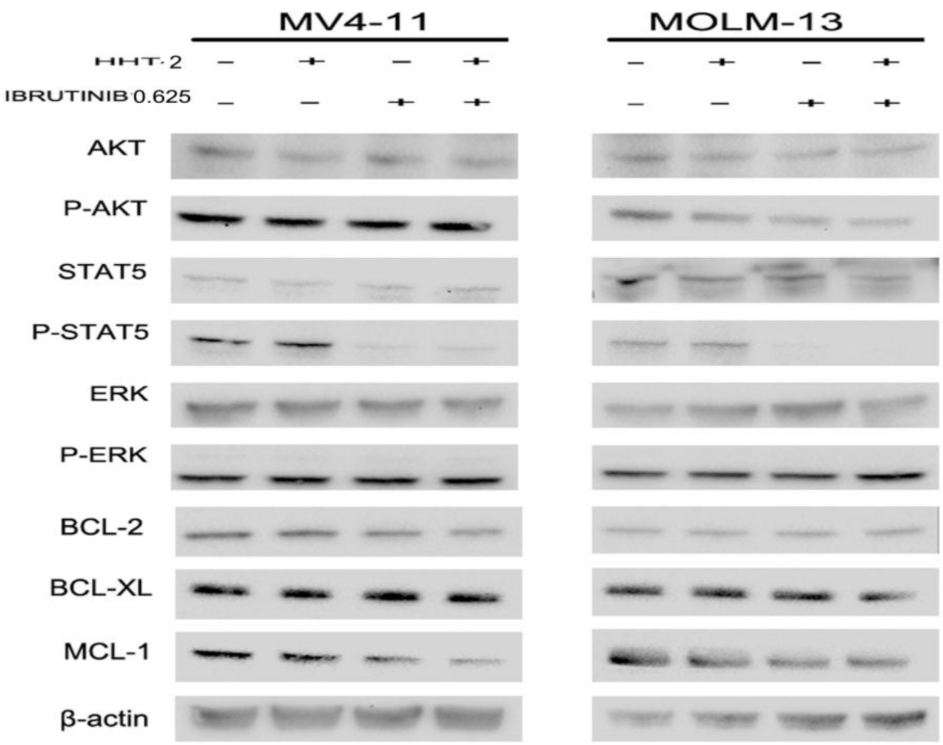

Supplementary Figure 1: The combination of 2 nM HHT and 0.625 µM ibrutinib down-regulated p-STAT5 and MCL-1.

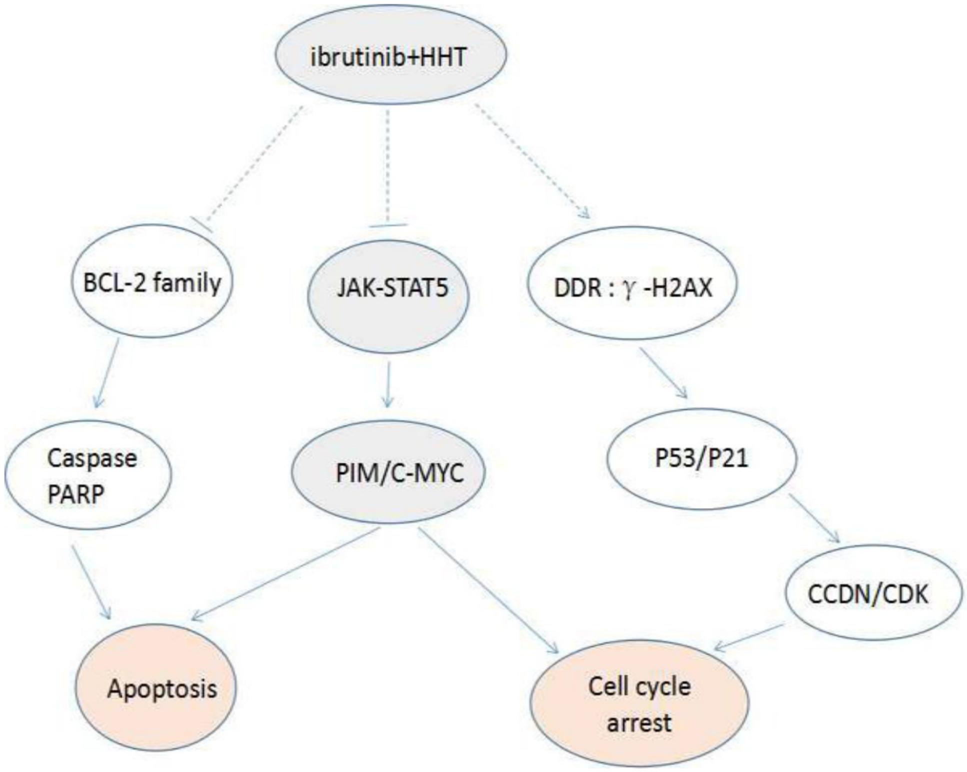

Supplementary Figure 2: The summarizing figure of the major findings and mechanisms.
